# Bloodstream Infections in Intensive Care Units: Microbial Spectrum and Antibiogram From a Tertiary Care Hospital in Central India

**DOI:** 10.7759/cureus.103389

**Published:** 2026-02-10

**Authors:** Pallavi S Tatte, Rohini Borkar, Yukta Chaudhari, Deepali Kawade, Pallavi Kadwe (Chintalwar), Neena Nagdeo

**Affiliations:** 1 Microbiology, NKP Salve Institute of Medical Sciences and Research Centre and Lata Mangeshkar Hospital, Nagpur, IND; 2 Biochemistry, NKP Salve Institute of Medical Sciences and Research Centre and Lata Mangeshkar Hospital, Nagpur, IND

**Keywords:** antibiogram, antimicrobial resistance, bloodstream infections, intensive care unit, sepsis

## Abstract

Background: Bloodstream infections (BSIs) are a major cause of morbidity and mortality among critically ill patients, particularly in intensive care units (ICUs). Increasing antimicrobial resistance among bloodstream pathogens further complicates management and limits effective empirical therapy.

Methods: This retrospective observational study was conducted in the Department of Microbiology at a tertiary care teaching hospital in central India. Blood culture samples received from various ICUs over a one-year period (July 2024 to June 2025) were analyzed. Organisms were identified using standard microbiological methods, and antimicrobial susceptibility testing was performed using the Kirby-Bauer disk diffusion method in accordance with Clinical and Laboratory Standards Institute guidelines.

Results: Out of 740 blood culture samples processed, 245 (33.10%) yielded positive growth. Gram-negative bacilli were the predominant isolates (44.89%), followed by Gram-positive cocci (35.92%) and fungal pathogens (19.19%). *Escherichia coli* and *Staphylococcus aureus* were the most common Gram-negative and Gram-positive isolates, respectively. Non-*albicans Candida* species constituted the majority of fungal isolates. Gram-negative organisms showed high resistance to cephalosporins and fluoroquinolones, while carbapenems, colistin, and tigecycline demonstrated the highest in vitro activity. All Gram-positive isolates were susceptible to vancomycin and linezolid, while *Candida* isolates exhibited notable azole resistance with preserved susceptibility to amphotericin B.

Conclusion: This study highlights the predominance of Gram-negative pathogens and a substantial burden of multidrug resistance among ICU-associated BSIs. Regular surveillance of bloodstream pathogens and institution-specific antibiograms is essential to guide empirical therapy and support antimicrobial stewardship efforts in critical care settings.

## Introduction

Bloodstream infections (BSIs) are a major cause of morbidity and mortality in hospitalized patients, particularly in tertiary care settings. When inadequately or inappropriately treated, BSIs can rapidly progress to sepsis, septic shock, multiorgan dysfunction, and death. Globally, sepsis affects an estimated 50 million individuals annually and is responsible for approximately 11 million deaths each year, accounting for nearly 20% of global mortality [1-3].

Clinically, BSIs are defined by the isolation of pathogenic microorganisms from blood cultures in the presence of compatible clinical features of infection. In patients admitted to intensive care units (ICUs), BSIs are associated with significantly higher mortality, prolonged length of hospital stay, and increased healthcare costs. Multiple studies have demonstrated that ICU-acquired BSIs substantially contribute to extended hospitalization and resource utilization [1,2].

BSIs may be categorized as community-acquired or healthcare-associated. Healthcare-associated BSIs occur more frequently in hospitalized and critically ill patients due to factors such as invasive procedures, indwelling intravascular devices, prolonged ICU stay, mechanical ventilation, and exposure to broad-spectrum antimicrobials [4]. Despite advances in intensive care management, life-support systems, and antimicrobial therapy, BSIs remain severe and potentially life-threatening conditions, with reported mortality rates ranging from 25% to 50% [4].

The cornerstone of BSI management includes early initiation of appropriate antimicrobial therapy, along with supportive treatment for associated complications such as septic shock and organ dysfunction [5]. Source control measures, including drainage of abscesses, surgical intervention, or removal of infected intravascular devices, play a critical role in selected cases [2].

In India, the burden of BSIs is particularly significant, with reported crude mortality rates ranging from 35.2% to 44.9%, which are considerably higher than those reported from developed countries such as the United States [2]. The increasing prevalence of antimicrobial resistance has emerged as a major challenge in the management of BSIs, limiting effective therapeutic options and complicating both empirical and definitive treatment strategies [6,7].

Antimicrobial resistance represents a major global public health crisis, with an estimated 4.95 million deaths associated with bacterial antimicrobial resistance in 2019, underscoring a substantial burden of morbidity and mortality worldwide, particularly in low-resource settings where resistance rates are often highest and treatment options are limited [8]. Institution-specific antibiograms, that is, cumulative summaries of antimicrobial susceptibility patterns, are essential tools for guiding empirical therapy, tracking evolving resistance, and informing targeted antibiotic selection, particularly in institutions where multidrug-resistant infections are common [9]. Antimicrobial stewardship programs (ASPs), which promote the optimal selection, dosing, and duration of antimicrobial therapy to improve clinical outcomes and minimize resistance, integrate antibiogram data into practice recommendations and have demonstrated importance in intensive care and other high-risk clinical settings [8,9].

Given the case fatality rates associated with BSIs in ICU patients, which range between 35% and 50%, continuous surveillance of bloodstream pathogens and their antimicrobial susceptibility patterns is essential. Institution-specific data on the epidemiology of BSIs and local antibiograms are crucial for guiding rational empirical therapy, optimizing patient outcomes, and reducing the burden of antimicrobial resistance [10]. 

International sepsis guidelines recommend obtaining blood cultures prior to initiating antimicrobial therapy in patients with suspected sepsis or septic shock to enable targeted therapy and antimicrobial de-escalation [11]. However, data on ICU-specific BSI epidemiology and antifungal resistance patterns from central India remain limited, highlighting the need for institution-level surveillance to guide empirical therapy.

Accordingly, the primary objective of this study was to determine the spectrum of pathogens causing BSIs in ICU patients and to analyze their antimicrobial susceptibility patterns in order to inform empirical treatment strategies and support antimicrobial stewardship in critically ill populations.

## Materials and methods

Study design and setting

This retrospective observational study was conducted in the Department of Microbiology of NKP Salve Institute of Medical Sciences and Research Centre and Lata Mangeshkar Hospital, a tertiary care teaching hospital in Nagpur, India. The study included blood culture samples received from various ICUs over a one-year period, from July 2024 to June 2025. Ethical approval for the study was obtained from the institute's Institutional Ethics Committee (approval number: NKPSIMS & RC and LMH/IEC/31/2024).

Sample collection and processing

All blood samples submitted to the bacteriology laboratory for the diagnosis of suspected BSIs during the study period were included. Samples were collected under strict aseptic precautions prior to the initiation of antimicrobial therapy. A volume of 5-10 mL of blood was collected from adult patients and 5 mL from pediatric patients [12]. Samples were inoculated into BacT/ALERT FA (adult) and PF Plus (pediatric) aerobic culture bottles (bioMérieux, Durham, North Carolina, United States) and processed using an automated blood culture system. Bottles were incubated for up to seven days [13]. Cultures showing automated growth signals were considered positive, while bottles without growth signals after seven days were reported as sterile for aerobic organisms. Positive broths were subcultured on blood agar and MacConkey agar and incubated under standard conditions.

For fungal isolation, blood samples from patients with suspected BSIs were separately collected in Brain Heart Infusion broth and incubated at 37°C. Subcultures were performed at 48 hours and on the seventh day on blood agar and MacConkey agar. Yeast isolates identified by colony morphology and Gram staining were subcultured on Sabouraud's dextrose agar. *Candida *species identification was performed using germ tube test, sugar assimilation, and fermentation tests, chlamydospore formation on corn meal agar, and colony colour on HiCrome *Candida* agar.

Identification of isolates and antimicrobial susceptibility testing

Bacterial isolates were identified using standard bacteriological and biochemical methods. Antimicrobial susceptibility testing was performed using the modified Kirby-Bauer disk diffusion method on Mueller-Hinton agar [14]. The results were interpreted in accordance with the Clinical and Laboratory Standards Institute (CLSI) guidelines [15]. Antibiotic discs were procured from HiMedia Laboratories, Mumbai, India.

Antifungal susceptibility testing was carried out using the HiComb minimum inhibitory concentration (MIC) method (HiMedia Laboratories, Mumbai) for amphotericin B, fluconazole, itraconazole, ketoconazole, and voriconazole [15,16]. Inocula were prepared in 0.85% saline, adjusted to a 0.5 McFarland standard, and inoculated onto RPMI-1640 agar supplemented with glucose. MICs were read at 24 and 48 hours after incubation at 35°C. *Candida albicans *ATCC 90028 and *Candida parapsilosis *ATCC 22019 were used as quality control strains. Interpretation was performed according to CLSI M27-A2 guidelines for fluconazole and itraconazole, while published arbitrary breakpoints were used for amphotericin B and ketoconazole [16,17].

Use of guidelines and diagnostic tools

All diagnostic methods, guidelines, and susceptibility testing standards used in this study, including the Kirby-Bauer disk diffusion method, CLSI antimicrobial and antifungal susceptibility guidelines, and standard microbiological identification techniques, are publicly available and free for academic and clinical use when appropriately cited. Commercially available diagnostic systems and reagents were used strictly according to manufacturers' instructions and are referenced for methodological transparency.

## Results

During the study period, a total of 740 blood culture samples from patients with suspected BSIs were processed. Of these, 245 samples yielded positive growth, resulting in an overall blood culture positivity rate of 33.10%.

Distribution of isolates

Among the 245 isolates, Gram-negative bacilli constituted the largest proportion (110 isolates; 44.89%), followed by Gram-positive cocci (88 isolates; 35.92%) and fungal isolates (47 isolates; 19.19%).

Among Gram-positive cocci, *Staphylococcus aureus *was the most frequently isolated organism (52 isolates; 21.22%), followed by coagulase-negative staphylococci (27 isolates; 11.02%) and *Enterococcus *species (9 isolates; 3.67%) (Table 1).

**Table 1:** Distribution of isolates obtained from blood culture samples (N=245)

Category	Isolate	Frequency (n)	Percentage (%)
Gram-positive cocci	Staphylococcus aureus	52	21.22
	Coagulase-negative staphylococci	27	11.02
	*Enterococcus *spp.	9	3.67
	Total	88	35.92
Gram-negative bacilli	Escherichia coli	33	13.47
	Pseudomonas aeruginosa	29	11.83
	Klebsiella pneumoniae	14	5.71
	Salmonella typhi	10	4.08
	Citrobacter freundii	9	3.67
	Acinetobacter baumannii	8	3.26
	Proteus mirabilis	7	2.86
	Total	110	44.89
Fungal isolates	Candida albicans	17	6.93
	Non-*albicans Candida*	30	12.24
	Total	47	19.19

**Figure 1:**
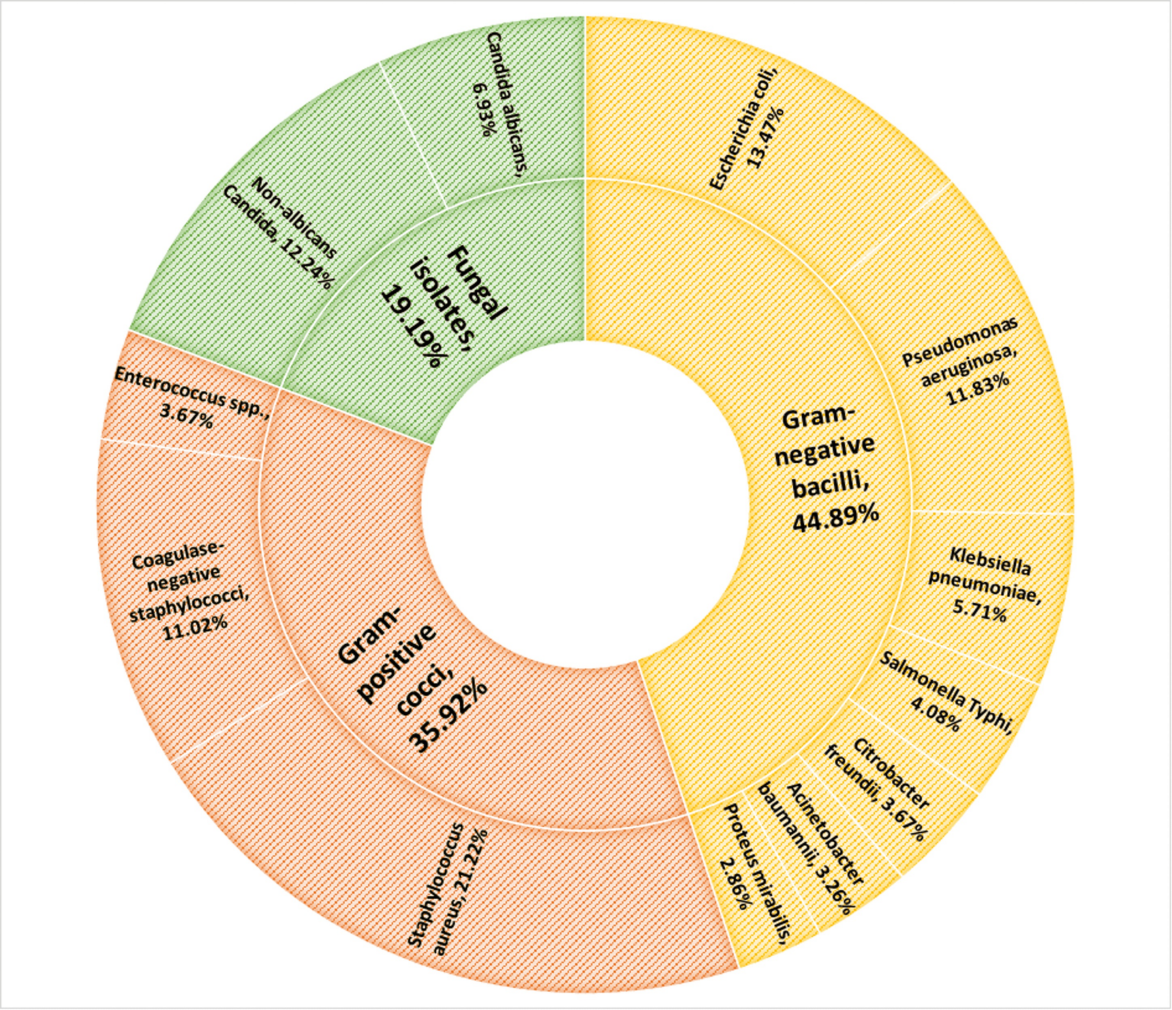
Distribution of isolates obtained from blood culture samples (N=245)

Gram-negative isolates were predominantly *Escherichia coli *(33 isolates; 13.47%) and *Pseudomonas aeruginosa *(29 isolates; 11.83%). Other Gram-negative organisms isolated included *Klebsiella pneumoniae *(14 isolates; 5.71%), *Salmonella typhi *(10 isolates; 4.08%), *Citrobacter freundii *(9 isolates; 3.67%), *Acinetobacter baumannii *(8 isolates; 3.26%), and *Proteus mirabilis *(7 isolates; 2.86%) (Table 1, Figure 1).

**Figure 2:**
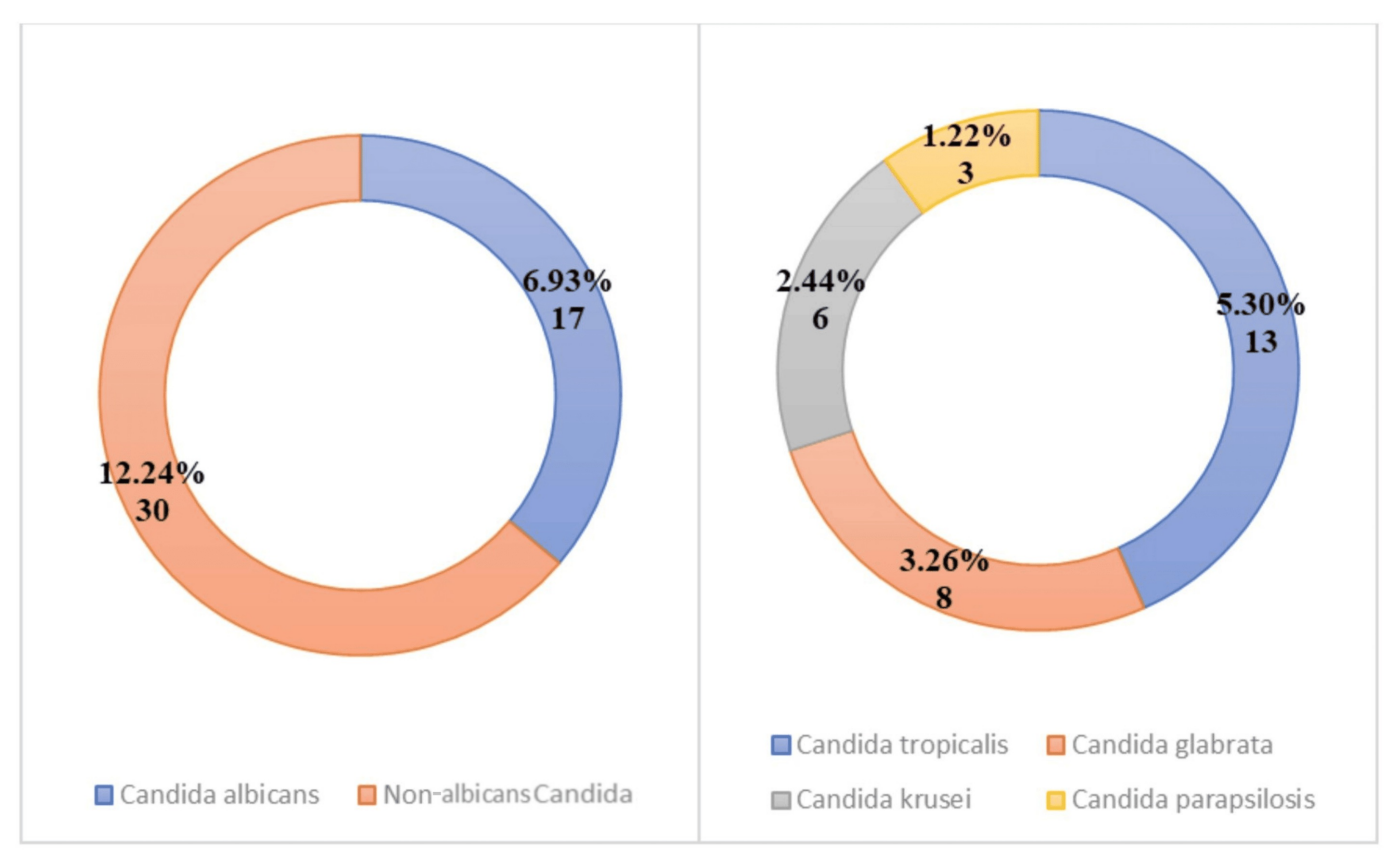
Distribution of fungi among isolates obtained from blood culture samples (N=245)

Fungal isolates accounted for 19.19% of all positive cultures. *Candida albicans *was isolated in 17 cases (6.93%), while non-*albicans Candida *species were isolated in 30 cases (12.24%). Among non-*albicans Candida*, *Candida tropicalis *was the most common species (13 isolates; 5.30%), followed by *Candida glabrata *(8 isolates; 3.26%), *Candida krusei *(6 isolates; 2.44%), and *Candida parapsilosis *(3 isolates; 1.22%) (Figure 2).

Distribution of samples across ICUs

The majority of blood culture-positive samples were obtained from the neonatal intensive care unit (NICU) (76 samples; 31.02%), followed by the pediatric intensive care unit (PICU) (38 samples; 15.51%), medical ICU (34 samples; 13.87%), surgical ICU (33 samples; 13.46%), respiratory ICU (32 samples; 13.06%), and intensive cardiac care unit (32 samples; 13.06%) (Table 2).

**Table 2:** Distribution of blood culture-positive samples across intensive care units (N=245)

Type of intensive care unit	Frequency (n)	Percentage (%)
Neonatal intensive care unit (NICU)	76	31.02
Pediatric intensive care unit (PICU)	38	15.51
Medical intensive care unit (MICU)	34	13.87
Surgical intensive care unit (SICU)	33	13.46
Respiratory intensive care unit (RICU)	32	13.06
Intensive cardiac care unit (ICCU)	32	13.06

Antimicrobial susceptibility pattern

Gram-Negative Isolates

Antimicrobial susceptibility testing of Gram-negative isolates demonstrated high resistance to third- and fourth-generation cephalosporins, including ceftazidime, ceftriaxone, and cefepime. Lower susceptibility rates were also observed for fluoroquinolones and trimethoprim-sulfamethoxazole across most Gram-negative organisms.

High susceptibility was observed to carbapenems, with *Escherichia coli*, *Klebsiella pneumoniae*, and *Pseudomonas aeruginosa *showing high sensitivity to imipenem and meropenem. Piperacillin-tazobactam also demonstrated good activity against most Gram-negative isolates. Tigecycline and colistin showed the highest susceptibility rates across all Gram-negative organisms tested, including non-fermenters such as *Acinetobacter *species (Table 3).

**Table 3:** Antimicrobial sensitivity pattern of Gram-negative isolates Values are expressed as number (%) of isolates susceptible to the respective antimicrobial agent. Antimicrobial susceptibility testing was performed using the Kirby-Bauer disk diffusion method and interpreted according to CLSI guidelines [14,15]. CLSI: Clinical and Laboratory Standards Institute

Antibiotic	*Escherichia coli *(n=33)	*Klebsiella *spp. (n=14)	*Pseudomonas aeruginosa *(n=29)	*Acinetobacter *spp. (n=8)	*Citrobacter freundii *(n=9)	*Salmonella typhi *(n=10)	*Proteus mirabilis *(n=7)
Amikacin	28 (84.8)	10 (71.4)	19 (65.5)	1 (12.5)	1 (11.1)	5 (50)	4 (57.1)
Gentamicin	20 (60.6)	7 (50)	11 (37.9)	0 (0)	1 (11.1)	5 (50)	5 (71.4)
Amoxicillin-clavulanate	20 (60.6)	11 (78.6)	12 (41.4)	3 (37.5)	3 (33.3)	8 (80)	4 (57.1)
Ceftazidime	15 (45.5)	3 (21.4)	10 (34.5)	0 (0)	0 (0)	8 (80)	2 (28.6)
Ceftriaxone	10 (30.3)	4 (28.6)	6 (20.7)	0 (0)	0 (0)	8 (80)	1 (14.3)
Cefepime	12 (36.4)	6 (42.9)	6 (20.7)	0 (0)	0 (0)	8 (80)	2 (28.6)
Piperacillin-tazobactam	30 (90.9)	12 (85.7)	27 (93.1)	7 (87.5)	7 (77.8)	10 (100)	7 (100)
Ciprofloxacin	0 (0)	3 (21.4)	13 (44.8)	0 (0)	1 (11.1)	10 (100)	4 (57.1)
Levofloxacin	10 (30.3)	4 (28.6)	12 (41.4)	0 (0)	1 (11.1)	7 (70)	2 (28.6)
Meropenem	30 (90.9)	13 (92.9)	27 (93.1)	7 (87.5)	7 (77.8)	10 (100)	7 (100)
Imipenem	28 (84.8)	13 (92.9)	27 (93.1)	7 (87.5)	7 (77.8)	10 (100)	7 (100)
Trimethoprim-sulfamethoxazole	28 (84.8)	1 (7.1)	3 (10.3)	0 (0)	0 (0)	6 (60)	0 (0)
Tigecycline	33 (100)	14 (100)	27 (93.1)	8 (100)	8 (88.9)	9 (90)	7 (100)
Colistin	33 (100)	14 (100)	28 (96.6)	8 (100)	9 (100)	10 (100)	7 (100)

Gram-Positive Isolates

Gram-positive cocci demonstrated high resistance to commonly used antibiotics such as ampicillin, erythromycin, clindamycin, and cotrimoxazole. Reduced susceptibility to gentamicin and cefoxitin was also observed among *Staphylococcus aureus *and coagulase-negative staphylococci.

All Gram-positive isolates, including *Staphylococcus aureus*, coagulase-negative staphylococci, and *Enterococcus *species, were uniformly susceptible to vancomycin and linezolid. Ampicillin-sulbactam demonstrated moderate activity against *Staphylococcus aureus *and coagulase-negative staphylococci (Table 4).

**Table 4:** Antimicrobial sensitivity pattern of Gram-positive isolates Values are expressed as number (%) of isolates interpreted as susceptible (S) according to CLSI criteria; isolates not categorized as susceptible were considered resistant (R) for reporting purposes. "–" indicates antibiotic not tested or not applicable. Antimicrobial susceptibility testing was performed using the Kirby-Bauer disk diffusion method and interpreted according to CLSI guidelines [14,15]. CLSI: Clinical and Laboratory Standards Institute

Antibiotic	Coagulase-negative staphylococci (n=27)	*Staphylococcus aureus *(n=52)	*Enterococcus *species (n=9)
Ampicillin	3 (11.1)	3 (5.8)	0 (0)
Gentamicin	8 (29.6)	10 (19.2)	–
Clindamycin	12 (44.4)	14 (26.9)	0 (0)
Cefoxitin	10 (37)	12 (23.1)	0 (0)
Ciprofloxacin	22 (81.5)	27 (51.9)	0 (0)
Cotrimoxazole	12 (44.4)	28 (53.8)	–
Erythromycin	12 (44.4)	14 (26.9)	0 (0)
Linezolid	27 (100)	52 (100)	9 (100)
Vancomycin	27 (100)	52 (100)	9 (100)
Ampicillin-sulbactam	25 (92.6)	22 (42.3)	2 (22.2)

The antifungal susceptibility profile of *Candida *isolates recovered from BSIs was evaluated (Table 5). Overall, fluconazole resistance was observed in 12 isolates (25.5%), with the highest resistance noted in *Candida albicans*, followed by *C. tropicalis*, *C. glabrata*, and *C. krusei*. Resistance to ketoconazole was also detected in 12 isolates (25.5%), predominantly among *C. albicans *and *C. tropicalis*, with additional resistance seen in *C. glabrata *and *C. parapsilosis*. Voriconazole resistance was identified in seven isolates (14.9%), with the highest proportion observed in *C. glabrata*, followed by *C. albicans*, *C. krusei*, and *C. parapsilosis*. Itraconazole resistance was seen in six isolates (12.8%), mainly among *C. albicans*, while lower resistance was observed in *C. tropicalis *and *C. glabrata*. Amphotericin B resistance was detected in four isolates (8.5%), primarily among *C. tropicalis *and *C. glabrata*. Notably, no resistance to amphotericin B was observed in *C. albicans *or *C. krusei*, and no resistance to itraconazole was detected in *C. krusei *or *C. parapsilosis*.

**Table 5:** Antifungal resistance pattern of Candida isolates from bloodstream infections Values are expressed as number (%) of isolates resistant to the respective antifungal agent. Antifungal susceptibility testing was performed using the HiComb MIC method and interpreted according to CLSI M27-A2 guidelines where applicable [16,17]. CLSI: Clinical and Laboratory Standards Institute; MIC: minimum inhibitory concentration

Antifungal agent	*Candida albicans *(n=17)	*Candida tropicalis *(n=13)	*Candida glabrata *(n=8)	*Candida krusei *(n=6)	*Candida parapsilosis *(n=3)
Fluconazole	4 (23.5)	3 (23.1)	2 (25)	2 (33.3)	1 (33.3)
Ketoconazole	4 (23.5)	4 (30.8)	2 (25)	1 (16.7)	1 (33.3)
Voriconazole	2 (11.8)	0 (0)	3 (37.5)	1 (16.7)	1 (33.3)
Itraconazole	4 (23.5)	1 (7.7)	1 (12.5)	0 (0)	0 (0)
Amphotericin B	0 (0)	2 (15.4)	1 (12.5)	0 (0)	1 (33.3)

## Discussion

BSIs remain a major cause of morbidity and mortality among critically ill patients, particularly those admitted to ICUs. In the present study, Gram-negative bacilli constituted the predominant group of bloodstream isolates (44.89%), followed by Gram-positive cocci (35.92%) and fungal pathogens (19.19%). This pattern is comparable to findings reported in several Indian ICU-based studies, including those by Gohel et al. and Gupta et al., where Gram-negative organisms were the leading cause of BSIs in hospitalized patients [18,19]. Similar dominance of Gram-negative pathogens in ICU infections has also been reported in antimicrobial resistance surveillance data from southern India, where non-fermenting Gram-negative bacilli and *Enterobacteriaceae* constituted the majority of isolates in ICU settings [20]. Al Sawafi et al. also reported a similar predominance of Gram-negative pathogens in ICU settings from the Middle East, where Gram-negative bacilli accounted for nearly two-thirds of ICU isolates, with *Klebsiella pneumoniae *emerging as the leading pathogen [21].

Among Gram-negative isolates, *Escherichia coli *(13.47%) was the most frequently isolated pathogen, followed by *Pseudomonas aeruginosa *(11.83%) and *Klebsiella pneumoniae *(5.71%). Similar distributions have been reported by Gohel et al., who identified *E. coli *as the most common Gram-negative bloodstream isolate, and by Gupta et al., who observed increasing isolation rates of *E. coli *and *Klebsiella *species over time [18,19]. International studies from high-income countries, including those by Friedman et al. and Nannan Panday et al., have demonstrated that *Escherichia coli *remains a leading cause of Gram-negative bacteremia; however, Gram-positive organisms continue to predominate overall in these settings [22,23]. In contrast, Moolchandani et al. observed a predominance of non-fermenting organisms such as *Pseudomonas *and *Acinetobacter *across ICU-associated infections, with *Enterobacteriaceae *more commonly associated with urinary and wound specimens than BSIs [20]. This difference may reflect variation in the type of infections studied, as the present study focused exclusively on BSIs, whereas their surveillance encompassed multiple healthcare-associated infection sites.

Gram-positive cocci accounted for 35.92% of isolates in the present study, with *Staphylococcus aureus *being the most common organism (21.22%). This finding is consistent with reports from Indian tertiary care hospitals, where *S. aureus *remains a major cause of ICU-associated BSIs [18,19]. In contrast, studies focusing on hospitalized and neonatal populations, such as that by Easow et al., have demonstrated a higher prevalence of coagulase-negative staphylococci, particularly in device-associated BSIs, highlighting the influence of patient population and ICU practices on pathogen distribution [6]. Moolchandani et al. also documented a substantial burden of methicillin-resistant *Staphylococcus aureus *(MRSA) in ICU settings, emphasizing the continued importance of resistant Gram-positive pathogens in critical care environments [20].

Fungal BSIs constituted a notable proportion of isolates (19.19%) in the present study, with non-*albicans Candida *species being more common than *Candida albicans*; these isolates were considered true pathogens when recovered from patients with clinical features of sepsis and predisposing risk factors (e.g., ICU stay, invasive devices), particularly when isolated from properly collected or repeat blood cultures, making contamination unlikely. Similar trends have been reported in Indian studies by Gupta et al. and others, indicating a shift toward non-*albicans Candida *species in ICU settings [19]. In comparison, Moolchandani et al. reported a lower overall proportion of *Candida *isolates among ICU infections, suggesting that candidemia burden may vary across institutions depending on patient case-mix, antimicrobial exposure, and invasive device utilization [20]. This contrast underscores the evolving epidemiology of ICU fungal infections and the need for local surveillance.

The antimicrobial resistance patterns observed in the present study are consistent with reports from Indian ICUs. Previous Indian studies, including those by Bohra et al. and Mathur et al., have documented high rates of MRSA, along with substantial resistance to commonly used antibiotics such as ampicillin, erythromycin, and clindamycin, while maintaining susceptibility to vancomycin and linezolid [24,25]. Similarly, Moolchandani et al. identified significant MRSA prevalence in ICUs but did not report widespread vancomycin resistance, aligning with our finding that glycopeptides and linezolid remain reliable agents against Gram-positive BSIs [20].

Gram-negative isolates in the present study demonstrated high resistance to third- and fourth-generation cephalosporins and fluoroquinolones, indicating a substantial burden of extended-spectrum beta-lactamase-producing organisms. Similar resistance patterns among Gram-negative bloodstream isolates have been reported in Indian ICU studies and national surveillance data, including reports by Mathur et al. and the Indian Council of Medical Research (ICMR) Antimicrobial Resistance Surveillance Network [25,26]. Moolchandani et al. also reported that more than half of Gram-negative ICU isolates were multidrug-resistant, with particularly high resistance among *Acinetobacter *and *Pseudomonas *species, and rising carbapenem resistance rates [20]. Carbapenems exhibited the highest in vitro activity against Gram-negative pathogens in this study; however, increasing reports of carbapenem resistance from other Indian centers highlight an emerging therapeutic challenge. The high resistance to third-generation cephalosporins and fluoroquinolones observed in our setting suggests these agents may be unreliable for empirical therapy in ICU-associated BSIs. Increasing reliance on carbapenems and colistin as last-line agents raises concern for further resistance selection, underscoring the need for strict antimicrobial stewardship and antibiogram-guided therapy. Comparable findings have been reported from ICUs in Latin America, where extremely high extended-spectrum beta-lactamase rates were observed among *Enterobacteriaceae*, with more than 80% of *Escherichia coli *and nearly 79% of *Klebsiella pneumoniae *isolates demonstrating extended-spectrum beta-lactamase production, correlating with marked resistance to third-generation cephalosporins and monobactams [27]. Worryingly, longitudinal ICU surveillance from Europe has demonstrated a progressive rise in colistin resistance among carbapenem-resistant *Acinetobacter baumannii *and *Klebsiella pneumoniae *bloodstream isolates, highlighting the erosion of last-line therapeutic options for severe Gram-negative infections [28].

The findings of the present study highlight the persistent burden of multidrug-resistant pathogens in ICU-associated BSIs. Consistent with previous Indian and international reports, infections caused by resistant organisms restrict effective empirical antimicrobial options and are associated with adverse clinical outcomes [10,11,19]. The ICU-focused antimicrobial resistance surveillance by Moolchandani et al. further reinforces that intensive care environments act as reservoirs for multidrug-resistant organisms due to high antimicrobial pressure, invasive device use, and critically ill patient populations [20]. Similar observations have been reported from ICUs in Latin America, where high multidrug resistance rates among Gram-negative bacilli were attributed to invasive procedures, prolonged hospitalization, and sustained exposure to broad-spectrum antimicrobials in critically ill patients [27]. In a tertiary care ICU study from Mexico, nearly three-quarters of Gram-negative isolates were multidrug-resistant, with particularly high multidrug resistance rates among *Escherichia coli*, *Acinetobacter baumannii*, and *Klebsiella pneumoniae*, underscoring the global scale of ICU-associated antimicrobial resistance [27]. Comparable ICU surveillance data from the Middle East have demonstrated that more than one-quarter of isolates were multidrug-resistant, with *Acinetobacter baumannii *being the most frequent multidrug-resistant organism and carbapenem-resistant *Enterobacteriaceae *representing some of the most challenging pathogens to treat [21]. Surveillance data from a tertiary hospital in Southern Europe have shown that nearly 70% of carbapenem-resistant Gram-negative bloodstream isolates were extensively drug-resistant (XDR), with the ICU accounting for the majority of these infections and *Acinetobacter baumannii *emerging as the predominant XDR pathogen [28]. Continuous local surveillance of bloodstream pathogens and their antimicrobial susceptibility profiles is therefore essential to guide empirical therapy, support antimicrobial stewardship programs, and reduce morbidity and mortality among critically ill patients. Such institution-specific surveillance data are critical for developing ICU-specific empirical treatment protocols and informing local antimicrobial stewardship policies in similar resource-constrained tertiary care settings.

The antifungal resistance pattern observed in this study highlights a concerning burden of azole resistance among *Candida *isolates causing BSIs. The distribution of resistance across multiple *Candida *species, particularly non-*albicans Candida*, mirrors trends reported in several Indian studies and reflects an evolving epidemiology of candidemia in tertiary care settings [29,30]. Fluconazole resistance was comparable to reports from other studies, and higher resistance to older azoles relative to voriconazole suggests selective pressure from widespread azole use. The comparatively preserved activity of voriconazole and amphotericin B is consistent with limited exposure and restricted use in clinical practice. Species-wise variability in resistance emphasizes the limitations of empirical antifungal therapy and reinforces the need for routine species identification and antifungal susceptibility testing to optimize patient management and curb the emergence of resistance. These findings have important implications for empirical antifungal selection in critically ill patients, where delays in initiating effective therapy are associated with increased mortality. The rising proportion of non-*albicans Candida *species with reduced azole susceptibility emphasizes the importance of early species identification and susceptibility-guided therapy.

This study has certain limitations. As a retrospective, single-center analysis, the findings may not be generalizable to other institutions or regions with different patient populations, antimicrobial prescribing practices, or infection control policies. Clinical outcomes such as mortality, duration of ICU stay, and prior antimicrobial exposure were not analyzed, limiting the assessment of the direct impact of specific pathogens and resistance patterns on patient outcomes. Despite these limitations, the study provides valuable institution-specific data on BSI epidemiology and antimicrobial resistance patterns in ICU settings, which are essential for guiding empirical therapy and antimicrobial stewardship.

## Conclusions

BSIs among ICU patients in this tertiary care hospital were predominantly caused by Gram-negative bacteria, followed by Gram-positive cocci and *Candida *species. *Escherichia coli *and *Staphylococcus aureus *were the most frequently isolated bacterial pathogens, while a substantial proportion of fungal infections were due to non-*albicans Candida *species, with notable resistance to commonly used azole antifungals. The antimicrobial susceptibility patterns revealed a high burden of multidrug resistance, particularly among Gram-negative organisms, with widespread resistance to cephalosporins and fluoroquinolones. Carbapenems, colistin, and tigecycline retained the highest in vitro activity against Gram-negative isolates, while vancomycin and linezolid remained uniformly effective against Gram-positive cocci.

These findings underscore the importance of routine blood culture surveillance and institution-specific antibiograms, along with antifungal susceptibility testing, to guide empirical therapy in ICU settings. Rational use of antimicrobials, supported by robust infection control measures and antimicrobial stewardship programs, is essential to reduce the emergence and spread of multidrug-resistant pathogens and improve clinical outcomes in critically ill patients.
